# Genetic diversity in clinical isolates of *Escherichia coli* by enterobacterial repetitive intergenic consensus (ERIC)–PCR technique in Sanandaj hospitals

**Published:** 2013-06

**Authors:** Rashid Ramazanzadeh, Serveh Zamani, Saman Zamani

**Affiliations:** 1 Cellular & Molecular Research Center and Microbiology Department, Faculty of Medicine, Kurdistan University of Medical Sciences, Sanandaj, Iran; 2 Student Research Committee of Medicine, Kurdistan University of Medical Sciences, Sanandaj, Iran

**Keywords:** *E. coli*, genetic diversity, ERIC–PCR, fingerprinting

## Abstract

**Background and Objectives:**

The *Escherichia coli* strains are greatly important in nosocomial and community acquired infections. The aim of this study was to determine the transmission of bacterial infections using genetic analysis.

**Materials and Methods:**

Two hundred and thirty *Escherichia coli* strains, isolated from different clinical samples, were characterized by enterobacterial repetitive intergenic consensus (ERIC)–PCR technique. The results and the similarity between the strains were determined on the basis of Jaccard similarity coefficient in the SAHN program of the NTSYS-pc software.

**Results:**

The ERIC–PCR profiles allowed typing of the 230 isolates into 205 ERIC-types which were grouped into twenty main clusters (C01–C20).The first group makes up 4.34% of the total isolates. Out of the 230 isolates, 34.2% belonged to D phylogenic group which were associated with extra-intestinal samples.

**Conclusion:**

Our results showed high diversity in *E. coli* isolates indicating low rate of hospital infection in our university hospitals. The majority of the isolates belonged to the D phylogenic group.

## INTRODUCTION

Bacterial genomes contain repeat sequences such as enterobacterial repetitive intergenic consensus (ERIC) sequence (1). These can be used as molecular biological tools to assess the clonal variability of many bacterial isolates including *E. coli* (2–4). The advent of molecular genetic techniques and their applications to microbial ecology has demonstrated that only a small proportion of natural microbial diversity has been discovered. Detailed information on the molecular genetics of previously uncharacterized micro-organisms may shed more light on the evolution of functionality of bacteria of a given species within its own habitat or in a foreign and hostile environment (5). REP-PCR is a genomic fingerprinting technique that generates specific strain patterns obtained by the amplification of repetitive DNA elements present along the bacterial genome (6–9). Enterobacterial repetitive intergenic consensus (ERIC sequences), also known as intergenic repetitive units, were initially reported *in Escherichia coli* and *Salmonella enterica* serovar Typhimurium. These sequences are now described in most bacterial species such as Enterobacteriaceae family as well as in *Vibrio cholera* (10, 11). The ERIC sequences have been located in intergenic regions as palindromes of 127 bps (11–14). Their copy numbers differ in different bacteria; e.g. 30 copies reported in *E. coli* K-12 and 150 in *S. enterica* serovar Typhimurium (15).

The aim of this study was to characterize the genetic diversity of *E. coli* isolates obtained from different clinical sources using ERIC-PCR. The degree of similarity between isolates was assessed by construction of a dendrogram which also enabled the comparison of clusters generated by analysis of samples from different sampling sites as well as assessment of the diversity of possible sources of contamination. The other specifications of the isolates were also considered for further evaluation.

## MATERIALS AND METHODS

### Study population and specimen types

This study was conducted at the School of Medicine, Kurdistan University of Medical science, Sanandaj, Iran. Consecutive, non-duplicate isolates of *E. coli* cells were collected from various specimens of patients who referred for Toohid and Beesat Hospitals. Most of the specimens were from the ICU, Infectious, Internal, Surgery, Women's and Pediatrics wards. Specimens included urine, wound, respiratory secretions, blood, cerebrospinal fluid, and others.

### Microbiological methods

All samples were routinely cultured on MacConkey and blood agar plates. Blood samples were cultured in Blood culture bottles. Isolates were identified at the species level using standard biochemical tests and microbiological methods such as colony types, motility, carbohydrate fermentation of glucose, lactose and sucrose, citrate assimilation, lysine decarboxylase, indole production, oxidase test, and catalase reaction.

### ERIC-PCR

*E. coli* isolates were fingerprinted using the enterobacterial repetitive intergenic consensus polymerase chain reaction (ERIC-PCR) (9). The primers used for the ERIC-PCR reaction were ERIC lR, 5'ATGTAAGCTCCTGGGGATTCAC3’ and ERIC2 5'AAGTAAGTGACTGGGGTGAGCG3’ (9).

DNA templates were prepared, purified and stored until needed at -20°C (16). ERIC-PCR reactions were performed in 25 µl volumes containing of 1 µl of each primer (final concentration 2 pmol/µl), 12.5 µl of Master Mix (Applied Biosystem) and 9.5 µl of deionized water. The ERIC-PCR reaction was as follows: initial denaturation at 95°C for 2min, with the next 35 cycles consisting of a denaturation step at 92°C for 30 s; annealing at 50°C for 1min; extension at 65°C for 8 min; as well as, a final extension step at 65°C for 8min and final storage at 4°C. The ERIC-PCR fragments obtained were examined by electrophoresis in 1.5% agarose gel. One-kb DNA markers (Fermentas Co.) were used and an electric field of 100 V were applied during electrophoresis for 45 minutes. The gels were subsequently stained for 30 min in a solution containing 0.5 mg of ethidium bromide per ml. (17).

### Computer-Assisted ERIC-PCR DNA Fingerprint Analysis

Gel images were captured using an electronic documentation system. ERIC-PCR fingerprints of amplified DNA fragments obtained by agarose gel electrophoresis were recorded. The positions of the bands on each lane and each gel were normalized using the 1 kb DNA ladder as an external reference standard. The presence of a given band was coded as 1 and the absence of a given band was coded as 0 in a data matrix and analyzed using the NTSYS-pc software (version 2.02 K, Applied Biostatistics, Inc., NY, USA). Dendrograms of dissimilarity were constructed for each case. The similarity between the strains was determined on the basis of the Jaccard similarity. The dendrogram was constructed on the basis of the averaged similarity of the matrix with the use of the algorithm of the Unweighted Pair-Group Method (UPGMA) in the SAHN program of the NTSYS-pc software. The nearest neighbor-joining clustering method has been used to show relations between similar groups.

### Phylogenetic grouping

Phylogenic groups were detected by PCR using primers for *chuA*, *yjaA*, and *tspE* genes (18).

## RESULTS

**Fig. 1:**
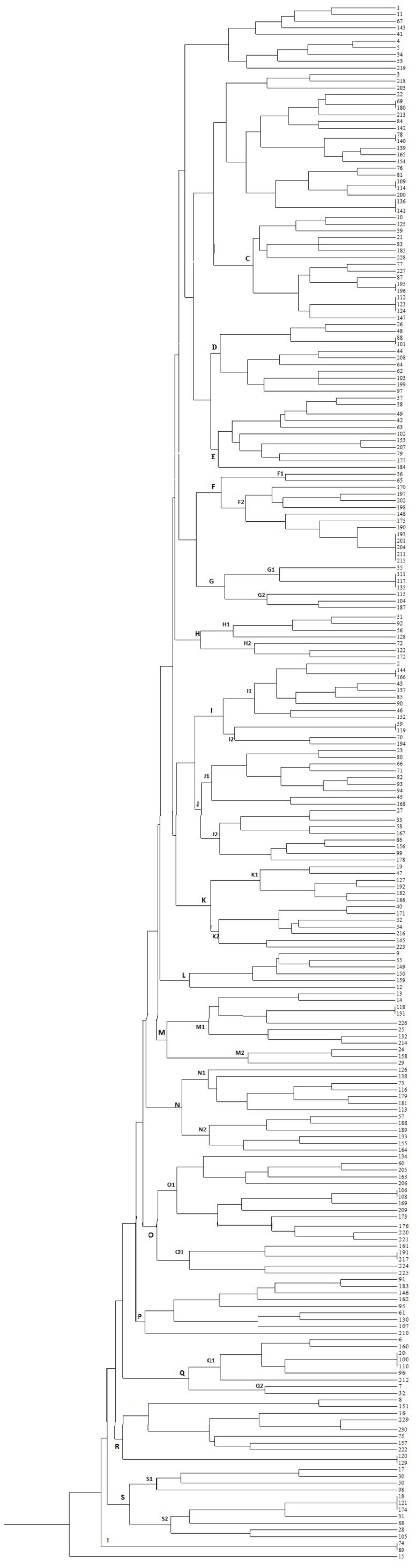
Dendrograms of genomic similarity of 230 *E. coli* strains in Sanandaj hospitals.

The genomic diversity analysis of 230 strains of *E. coli* has been carried out using the ERIC-PCR fingerprinting method with ERIC-type primer. The electrophoretic profiles of the DNA fragments obtained after PCR amplification using specific primers for ERIC sequences were determined for the *E. coli* isolates. The ERIC–PCR profiles allowed differentiation of the 230 isolates into 205 ERIC-types which were grouped into twenty main clusters (C01–C20) (Table 1). Complex patterns of fingerprints have been obtained for all strains. Generally, the electrophoretic analysis of the PCR reaction products has revealed that the number of bands in particular electrophoretic paths ranged from 6-15. The sizes of the PCR products ranged from slightly less than 100 bp to about 1400 bp. Products ranging from 200-800 bp were encountered more routinely. The dendrogram has grouped the 230 strains of *E. coli* into ten similarity groups. Isolates of the first group make up 4.34% of the totalisolates (Fig. 1). Their property similarity is 59%, as shown in (19) (Table 1). Characterization of 18 patterns and genetic diversity of each pattern that contain more than one isolate is illustrated in Table 2. Out of the 230 isolates, 34.2% of them belonged to D phylogenic group that were associated with extra-intestinal samples.

**Table 1:** Clustering and similarity of *E. coli* isolates and Number of strains in each cluster.

Clusters	Dendrogram similarity in clusters	Number of strains in each cluster
C01	59%	10 4.34
C02	57%	21 9.13
C03	65%	16 6.95
C04	58%	11 4.78
C05	55%	11 4.78
C06	56%	14 6.08
C07	56%	7 3.04
C08	50%	7 3.04
C09	57%	13 5.65
C10	49%	17 7.39
C11	53%	13 5.65
C12	47%	6 2.60
C13	40%	11 4.78
C14	45%	13 5.65
C15	35%	18 7.82
C16	35%	9 3.91
C17	51%	9 3.91
C18	30%	10 4.34
C19	33%	11 4.78
C20	100%	2 0.87

**Table 2:** Genetic diversity and characterization of 18 patterns of 230 *E. coli* clinical isolates.

pattern		Clusters	groups	Specimens					Hospital ward						ESBL	

	No.			Blood	CSF	lung discharge	Urine	Wound	Women's	Out patients	ICU	Surgery	Pediatrics	Internal	Yes	No
**SZ15**	2	C02	B2	1		1					1	1			2	
**SZ19**	2	C02	B2	1			1		1		1					2
**SZ25**	2	C02	D				2			2						2
**SZ27**	2	C02	B1				2		1	1					2	
**SZ40**	2	C03	D			1	1				1			1		2
**SZ41**	3	C03	A				3		2	1					3	
**SZ45**	2	C04	D				2			1		1				2
**SZ53**	2	C05	B1	1			1		1				1		2	
**SZ72**	5	C06	A		1		4			1	2		2			5
**SZ74**	3	C07	A			1	2		1	1	1				2	1
**SZ86**	2	C09	B2			1	1			1	1					2
**SZ93**	2	C09	B1				2			1				1	2	
**SZ134**	3	C13	B2			1	1	1	1	1	1					3
**SZ168**	2	C15	D			1	1						1	1		2
**SZ182**	3	C17	D			1	2		2		1					3
**SZ185**	2	C17	D			1	1				1			1	2	
**SZ194**	2	C18	B1				2			2						2
**SZ204**	2	C20	D				2			1			1			2
**Total**	43			3	1	8	30	1	9	13	10	2	5	4	15	28

## DISCUSSION

This is the first study in this region to elucidate the genetic diversity of *E. coli* clinical isolates. There are several methods for determination of bacterial transmission trace in the community and hospitals. ERIC-PCR has been used by several studies and for different bacterial isolates including *Pseudomonas aeruginosa*, *Heamophilus spp*. (20), *Vibrio cholera* (21), *Corynebacterium* (22), *Salmonella spp*. (23), *Streptococcus* (24), and other bacterial strains (25, 26). ERIC-PCR typing method showed 205 patterns for 230 clinical isolates. One hundred eighty-seven (81.3%) of the isolates displayed a single profile; whereas, 43 (18.7%) of them showed shared patterns which is indicative of similar origin of dissemination. ERIC-PCR profiles did not demonstrate genetic relatedness between 187 *E. coli* strains. Therefore, infections caused by most of our university hospital isolates were not clonally spread and resulted from individual infections. Eighteen profiles each containing 2 to 5 isolates showed similar genotype which indicate clonal spreading of bacteria in hospital wards. Similar results were obtained for *E. cloacae* isolates of nosocomial origin by ERIC-PCR technique (27).

Molecular epidemiology using ERIC-PCR fingerprinting patterns showed that isolates in cluster exhibit similar profiles, which demonstrate the clonal transmission of bacteria in hospital environments. In addition, the bacterial patterns were further divided into twenty main clusters (C01–C20) with the C02 cluster being the major one. Clonal transmission of *A. baumannii* has been evidenced by ERIC-PCR DNA fingerprinting patterns in multi-drug resistant isolates (28). In another studies, *E. coli* and *K. pneumoniae* nosocomial outbreaks in Novosibirsk Regional Hospital were studied by ERIC-PCR (29).

The aim of this study was to show the genetic relationship in isolates and their hospital transmission pattern. Most of the isolates show unique patterns which indicate that the rate of nosocomial infections are very low in Sanandaj University hospitals. The remaining patterns showed similarity in most characteristics. The strains in this study presented similar antibiotic resistance patterns especially in regards to extended spectrum beta lactamase (ESBLs). We reported the rate of ESBL production in *E. coli* isolates in previous reports (30–36). Phylogenetic group D was the most prevalent (34.22%) in our results. Distribution of the other groups was as follows: B2 (32.89%), A (21.78%), and B1 (11.11%). As reported previously, the phylogenetic groups B2 and D of *E. coli* strains are mostly associated with extra-intestinal infections; whereas, groups A and B1 bacteria are considered to be commensal isolates (37, 38).

In conclusion, ERIC-PCR fingerprint types suggested considerable diversity in *E. coli* isolates which may be due to the low rate of hospital infection in our university hospitals. The rate of hospital infection is very low and most isolates belonged to the D phylogenetic groups that were associated with extra-intestinal samples.
